# COVID-19 and Climate Change: Re-thinking Human and Non-Human in Western Philosophy

**DOI:** 10.1007/s11673-023-10277-0

**Published:** 2023-07-11

**Authors:** G. Lloyd

**Affiliations:** Emeritus Professor in Philosophy, University of New South Wales, New South Wales, NSW Australia

**Keywords:** COVID-19, Climate change, Descartes, Spinoza

## Abstract

The pre-conditions and the effects of the COVID-19 pandemic are inter-connected with those of climate change, prompting reflection on how to re-think the relations between human and non-human on a changing planet. This essay considers that issue with reference to the contrasts between the philosophies of Descartes and Spinoza, who offered radically different approaches to the conceptualization of human presence in Nature.

The shocking immediacy of impacts of the coronavirus pandemic has brought a heightened awareness of other crises with which it is inter-connected. As the economic historian Adam Tooze has observed, in his book *Shutdown,* the virus has laid bare the historical transformation of Nature and its implications for the future of humanity. Even those most sheltered from its effects have been stripped of the illusion that the “Anthropocene” can be regarded merely as an abstract intellectual proposition (Tooze 2021).

The pre-conditions of the pandemic, as well as its effects, are inter-related with those of climate change. Both crises reflect the increasing proximity of human beings and animal habitat, exacerbated by de-forestation. Both are enmeshed with mass displacements of people within nations, as well as with the flow of refugees across national borders. The crises have also in common that they pose a strategic dilemma: although their effects are immediate and local, dealing with them demands long-term global strategies enacted across the borders which separate nation-states.

While focusing attention directly on virus transmission across species, the on-going pandemic has thus cast a spotlight also on connected issues. Underlying them are deep conceptual questions which centre on how best to now *think* the newly visible inter-relations of *human* and *non-human* under conditions of environmental change.

Western philosophy has a long history of regarding humanity as having a special status within Nature. That is not in itself surprising. After all, theorizing the nature and conditions of the good human life is itself an achievement of reasoning—a capacity traditionally regarded by philosophers as a distinguishing feature of the human species. However, there is a range of ways in which content has been given to that supposed special position of human thought processes within the totality of being. Not all those ways of thinking are appropriately seen as complicit in the historical transformation of Nature. Yet the cumulative force of past philosophy has left an enduring presumption—not just that human beings are endowed with superior intellectual capacity but that they are justified in claiming rightful dominance over non-human parts of Nature.

Seventeenth and eighteenth century philosophers are strongly associated with that mentality of human supremacy. The era now known as “The Enlightenment” is credited with facilitating the advance of modern science and the development of liberal political institutions. However, Enlightenment thought is also increasingly implicated in the shaping and sustaining of mind-sets which legitimated and fostered European colonialist expansion and its destructive exploitation of natural resources and human labour. Yet, although the history of Western philosophy has indeed contributed to the conceptual underpinnings of attitudes of supremacy, it can also offer resources for contemporary re-imagining of the relations between human and non-human.

Among the most notorious of philosophical views seen as endorsing human supremacy within Nature is the strong version of *dualism* associated with the French seventeenth century philosopher, René Descartes (1596–1650). In his best-known work, the *Meditations*, he argued that human minds are self-contained *intellectual substances,* which are radically separated from matter by their distinctive capacity for *Thought* (Descartes 1984). Such minds were nonetheless treated by Descartes as causally interacting with human bodies across that gulf of difference. Non-human bodies—lacking causal connection with non-material *minds*—were treated in his philosophy as insentient automata.

Descartes’s actual position on the interactions of minds and bodies was subtler than the simplified version of *dualism* with which he has been identified in popular commentary. However, what is relevant here is neither the truth of his theory of mind–body relations nor the accuracy of current interpretations of his views. What is significant is the lasting power of the way of imagining human presence in Nature which his name has come to epitomize. The view of humanity as essentially separate from the rest of Nature has both nourished and been reinforced by a wider mentality of supremacy, dominance, and exploitation, which lingers in collective imagining of what it is to be human. That mentality has increasingly come under challenge amidst the intellectual and emotional impacts of COVID-19 and climate change.

Breaking the grip of the “Cartesian” model can involve de-stabilizing shifts of thought. It demands more than re-iteration of superficial mantras of humankind being “part of Nature”. It calls for deep re-imagining of what it is to be human—informed by contemporary scientific understanding of the inter-dependence of *human* and *non-human.* In the context of such re-thinking, it can be helpful to look to other, less familiar strands in the history of philosophy, which offered alternative ways of thinking of human presence in the world.

Another seventeenth century philosopher, Benedict de Spinoza (1632–1677), argued strongly, in his *Ethics,* against Descartes’s view of the nature of human minds and of their relations with the rest of Nature (de Spinoza, 1985). He ridiculed the notion that human beings inhabited, as it were, a separate, exclusive zone of autonomy within the wider domain of Nature. Rather, he insisted, they are immersed in Nature and subject to the same forces that govern the whole.

Unlike Descartes, Spinoza regarded animals as having their own powers and pleasures, rather than being insentient automata. Nor did he think of what is non-human as occupying a lower position on a God-given scale of perfection. He did nonetheless believe that human beings are justified in using what is non-human for their own benefit, repudiating concern for the suffering of animals.

From a contemporary perspective, what is interesting here is the very different way in which Spinoza justified human indifference to the well-being of other species. For him, the special position of humankind within Nature was not grounded in the status of minds as individual *intellectual substances.* Minds, he thought, are essentially *embodied* and hence inter-dependent with all other finite things. In his terms, human minds are *modes* of the one unique *Substance,* which he calls *God-or-Nature.* Within that totality of being, embodied human minds find their own distinctive well-being in connecting and collaborating with one another —a shared thriving, suffused with joy in the recognition of commonality. In his political writings, he describes that reciprocal recognition of sameness as “loving *kind*-ness”*—*evoking an experience of affective affinity.

How might the contrasts between Cartesian *dualism* and Spinozist *embodiment* bear on contemporary efforts to re-think the relations between human and non-human? Although Spinoza did not see those relations as warranting concern for animal well-being, his way of thinking of human presence in Nature is more amenable than Descartes’s to revision in the light of increased scientific understanding—both of commonalities between human and non-human life, and of their inter-relations in a shared environment. The Cartesian model is at odds with current recognition of the extraordinary collective intelligence shown in non-human adaptation to environmental change. Yet that model lingers, for example in the persistence of the conviction that only what is human—or at least human-like—warrants the ascription of *Mind* or *Thought,* as distinct from mere drive or instinct.

One significant rhetorical strategy in contemporary efforts to re-think human presence in Nature has been the resort to a consciously inclusive locution of the *more-than-human*. The Australian social geographer, Lesley Head, writing in her book, *Hope and Grief in the Anthropocene,* has explicated that locution as directed towards acknowledging the pervasiveness of human influence, while stressing also its inter-dependence with non-human existence—both animate and inanimate. The strategy challenges the assumed centrality of human presence in Nature, re-figuring humankind as just one among other species—though one that is also capable of wreaking harm on the inter-dependent whole (Head 2016).

Such rhetorical strategies serve as reminders that the human and the non-human have not always been construed in practice as in a binary opposition. Human beings have ever shaped—and been shaped by—the rest of Nature. Yet, in challenging the separateness of humankind, talk of the “more-than-human” raises unresolved issues about how exactly to conceptualize the *human* within the shared domain of Nature. Is it really just one-among-others? Or is it a first-among-equals? And what of human thought itself? Is it to be included in the totality of the more-than-human? Or is it better seen as the encompassing frame within which the totality of Nature is unified?

Here, the contrasts between the familiar Cartesian and the less familiar Spinozist ways of imagining human thought can help clarify what is at stake. The Cartesian model postulates a fundamental separateness of *Thought* from the rest of Nature—a self-sufficient standpoint from which thinking minds view the world. On the Spinozist alternative, human minds are themselves inherently embodied. Hence they know Nature only from within—through the effects of other bodies on their own. Human thought is thus itself immersed in the totality of being—constantly affected by the rest of the totality.

The ramifications of such a re-positioning of human thought are yet to be fully explored. Spinoza’s own articulations of his alternative model are offered within a complex metaphysical system in which *Thought* and *Matter* are *Attributes* of the one *Substance—God-or-Nature.* Not all of that system can be readily appropriated into a contemporary way of thinking of the place of humankind in Nature. However, the significance of his philosophy for now re-thinking the relations between human and non-human does not lie in championing one philosophical system against another. It lies in recognizing the contingency and changeability of the familiar model of *separateness* which has shaped—and continues to reinforce—assumptions of human supremacy over the non-human.

Understanding something of the history of conflicting models of thought in Western philosophy can contribute to clarifying some of the conceptual complexities which have been thrown up by the inter-connections of a global pandemic and climate change. It can open up fresh possibilities for reflection, under those changing conditions, on what it is to be human.

There remains, however, something that seems unavoidable about giving human thought a special significance within a newly visible more-than-human totality. It is from the perspective of human observation—using categories articulated within human thought — that relations across species are theorized. The capacity to think “theoretically” remains a distinctive feature of human thought—even when that thinking apprehends with awe the subtleties of collective intelligence among non-human species. The challenge here is not to escape that human framing but rather to better understand its nature and its implications.

The COVID-19 pandemic has prompted not only re-thinking the relations between human and non-human but also reflection on the commonalities of being human. There is some irony, then, in the fact that national responses to the epidemic have also made visible the structural inequalities which divide human collectivities—both within and between nation-states. Contemporary global inter-connections, which have fostered the transmission of the virus, have also facilitated the extraordinary achievement of rapid vaccine development through collaboration across borders—with potential benefit to *all* human beings. Yet political responses to the crisis have been largely shaped by an ethos of *separateness*: of border enforcement, containment, and exclusion—exacerbating existing inequalities.

The combined impact of the pandemic and climate change have brought a heightened awareness of the inter-dependence of human and non-human existence and thriving on a changing planet. Perhaps deeper understanding of those inter-connections may yet bring also a stronger sense of what is distinctive about being *human* under those changing conditions. Spinozist loving-*kind*ness may serve humanity better than destructive illusions of supremacy over the rest of Nature. Out of the immediacy of grief at all that is lost—human and non-human—there may yet come hope of salvaging a shared future.
